# The Role of Urea Cycle Functional Studies in Preclinical Research

**DOI:** 10.1002/jimd.70225

**Published:** 2026-07-15

**Authors:** Nathan Breuillard, Nadia Zürcher, Erica Faccin, Kim L. Stocker, Martin Poms, Johannes Häberle

**Affiliations:** ^1^ Division of Metabolism and Children's Research Centre (CRC) University Children's Hospital Zurich Zurich Switzerland; ^2^ Division Clinical Chemistry and Biochemistry University Children's Hospital Zurich Zurich Switzerland

## Abstract

Inborn errors of metabolism affecting the urea cycle are rare severe conditions caused by impaired nitrogen detoxification, leading to hyperammonemia and neurological morbidity across a broad clinical spectrum. Current biochemical diagnostics largely rely on quantification of metabolites. While indispensable for diagnosis and acute management, these snapshot readouts are highly dependent on external factors and often fail to reflect urea cycle function in metabolically compensated states, limiting their usefulness for disease stratification, therapeutic monitoring, and preclinical evaluation of treatments. Here, we reflect on the rationale, development, and application of a functional ureagenesis assay based on administration of [^15^N]‐labeled ammonium chloride, enabling quantitative assessment of total in vivo urea cycle function. This assay development was prompted by discordant biochemical and phenotypic findings in a gene therapy‐treated mouse model of ornithine transcarbamylase deficiency where supraphysiological enzyme activity did not translate into clinical improvement. We demonstrate that the [^15^N]‐isotope based ureagenesis assay is applicable to in vivo models and enables detection of functional correction of the urea cycle. Importantly, we show its applicability in metabolically stable individuals affected by urea cycle disorders, where classical biochemical markers are often normal despite significant enzyme or transporter impairment. Together, these data establish the [^15^N]ammonium chloride‐based functional ureagenesis assay as a robust and sensitive tool for quantifying urea cycle function. This approach offers clear advantages for preclinical research and translational studies, particularly in the evaluation of novel therapeutic strategies where traditional biomarkers lack discriminatory power.

## 1 Introduction

Inborn errors of metabolism (IEMs) affecting the urea cycle (urea cycle disorders, UCDs) represent a group of rare (cumulative incidence around 1 in 35 000 [1]) but severe disorders characterized by impaired nitrogen/ammonia detoxification [2, 3]. The clinical spectrum ranges from severe neonatal hyperammonemia to compensated adult‐onset disease with subtle neurocognitive manifestations [4, 5, 6]. Traditionally, the biochemical evaluation of UCDs relies on the quantification of metabolites such as ammonia, glutamine, citrulline, arginine, argininosuccinate, and urinary orotic acid [7]. These measurements are indispensable for a rapid diagnosis and acute management; however, they are usually taken at single timepoints and therefore provide only a snapshot of a highly dynamic metabolic pathway.

In compensated states, metabolite levels may be found within the normal range despite a severe defect of one of the six enzymes or two transporters of the cycle. This dissociation between metabolite concentrations and pathway function limits the ability to assess disease severity, monitor therapeutic efficacy, or evaluate preclinical interventions [8]. The need for reliable biomarkers is particularly important for evaluation of novel therapies such as small‐molecule modulators, mRNA therapy, gene replacement, and genome editing, which are commonly tested first in clinical trials in metabolically stable individuals.

The present work originated from a real‐world laboratory observation in the *spf* ^ash^ mouse, a widely used model of the most common UCD, ornithine transcarbamylase (OTC) deficiency [9]. Despite supraphysiological OTC enzyme activity in liver homogenate following gene delivery, no phenotypic improvement was observed [10]. This highlighted the limitations of classical biochemical assays and motivated the development of a functional assay, thereafter referred to as ureagenesis assay, capable of quantifying total in vivo urea cycle functionality in metabolically stable situations [11, 12].

Here, we describe the rationale, development, and application of a [^15^N]‐labeled ammonium chloride‐based ureagenesis assay in preclinical models and human subjects. We demonstrate its utility in distinguishing compensated mutant from wildtype animals, in evaluating therapeutic interventions, in characterizing cellular models, and in assessing urea cycle function in metabolically stable patients where classical biomarkers fail.

## 2 Limitations of Classical Biochemical Assessment of Urea Cycle Function

The *spf* ^ash^ mouse is one of the most widely used preclinical models for OTC deficiency [9]. These animals harbor the missense variant *Otc*‐Arg129His, which effectively leads to mis‐splicing and a dysfunctional OTC due to the insertion of 16 amino acid residues, eventually resulting in approximately 5%–10% residual hepatic OTC activity with broad intra‐ and interanimal variability [9, 13, 14, 15]. These mice exhibit biochemical abnormalities under stress or high‐protein challenge but remain largely compensated under standard laboratory conditions [13].

In an effort to restore OTC function, *spf* ^ash^ mice underwent hydrodynamic tail vein injection of a vector encoding a codon‐optimized murine *Otc* gene. Several weeks after treatment, mice were sacrificed and hepatic OTC activity was quantified. Surprisingly, enzyme activity in liver homogenate exceeded 100% of wildtype levels, a finding consistent across multiple animals and independent experiments [10]. Despite this clear increase in measured enzyme activity, no improvement in clinical and biochemical phenotype was observed. Histological analysis revealed that the naked DNA vector localized almost exclusively to the pericentral hepatocytes, an anatomical region within the liver with no expression of the urea cycle which is primarily located in periportal liver cells [16, 17]. Since ammonia detoxification is tightly regulated by this zonation, any supraphysiological OTC activity in the pericentral region does not translate into increased ureagenesis. This observation underscored a limitation relevant for all UCDs: enzyme activity assays in liver homogenates do not necessarily reflect urea cycle functionality.

Further biochemical characterization of *spf* ^ash^ mice revealed profound limitations in the use of classical metabolite measurements to assess urea cycle function. Although ammonia is the most widely used biochemical marker in both clinical and preclinical settings, its diagnostic value in compensated *spf* ^ash^ mice proved to be extremely limited. When measured at 5 weeks of age under standard husbandry conditions, blood ammonia concentrations in mutant animals were essentially indistinguishable from those of wildtype littermates [13]. Ammonia concentrations varied substantially depending on the site of blood sampling. Tail‐vein sampling consistently yielded lower values than samples obtained from the saphenous vein, and both were influenced by the degree of restraint and handling stress experienced by the animal. In addition, ammonia exhibited a pronounced diurnal rhythm, with higher concentrations observed at the beginning of the light phase in both wildtype and mutant mice. This circadian fluctuation further reduced the ability of single time point measurements to reflect underlying metabolic impairment. Collectively, these observations demonstrate that ammonia levels in *spf* ^ash^ mice are strongly influenced by sampling conditions and physiological state and not only by the presence of OTC deficiency itself [13].

Orotic acid, another commonly used biomarker in UCDs, showed similar limitations. Although group‐wise comparisons revealed statistically significant differences between wildtype and *spf* ^ash^ mice, the degree of overlap between individual animals was substantial. Many mutant mice exhibited orotic acid concentrations that fell directly within the wildtype range. Moreover, longitudinal sampling over several weeks revealed marked intra‐animal variability, with orotic acid levels fluctuating widely even in the absence of dietary or environmental perturbations [13]. This instability rendered orotic acid poorly reproducible as a readout of disease severity.

Taken together, these findings highlight a fundamental limitation of classical metabolite‐based assessment in preclinical models of UCDs. Both ammonia and orotic acid, despite their central role in clinical diagnostics, lack the sensitivity and specificity required to distinguish compensated mutant animals from their wildtype counterparts. Their high susceptibility to sampling conditions, physiological fluctuations, and intra‐individual variability despite genetic homogeneity and standardized environment severely constrains their utility for evaluating therapeutic interventions or for quantifying residual metabolic function. These shortcomings underscore the need for a pathway‐level functional assay capable of capturing true urea cycle function rather than relying on a snapshot of metabolite concentrations. All this finally motivated the development of a functional assay to quantify total ureagenesis.

## 3 Development of a Novel Functional Ureagenesis Assay

The urea cycle detoxifies ammonia by converting it into urea through a series of enzymatic steps. Administering [^15^N]‐labeled ammonium chloride provides a direct substrate for the pathway. After absorption in the small intestine, labeled ammonium is transported via the portal vein to periportal hepatocytes, where it enters the urea cycle and is eventually incorporated into [^15^N]urea. The mass difference between labeled and unlabeled urea (61 vs. 60 g/mol) is detectable using high‐resolution mass spectrometry (HR‐MS). The newly developed assay described herein follows the same principle that was established earlier by the pioneering work of Yudkoff et al., at that time using less sensitive technology [18].

**FIGURE 1:**
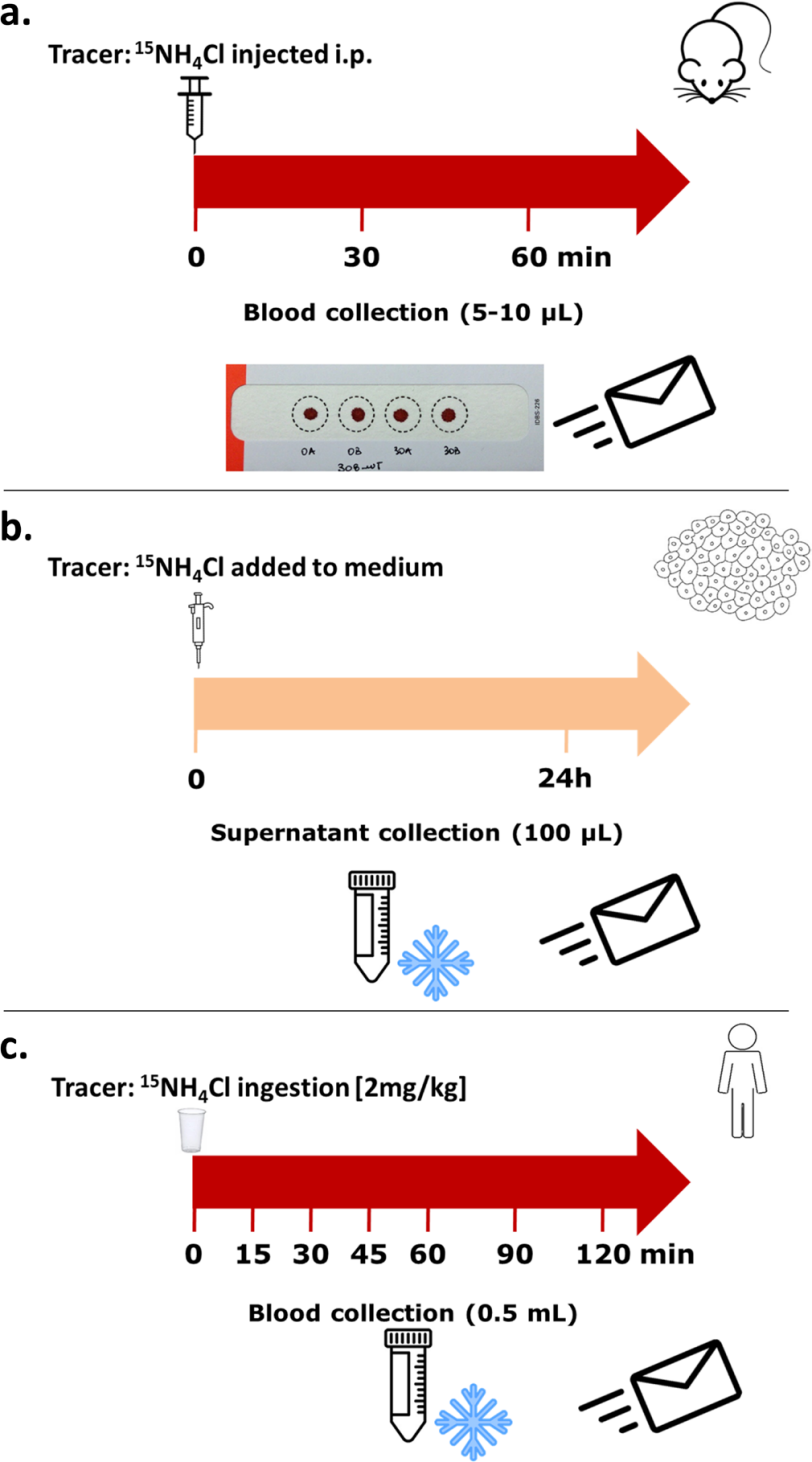
The figure shows in a simplified way the practical aspects of the ureagenesis assay when performed in mice (a) or cells (b); for comparison, the assay procedure is also shown for humans (c, modified from b, in [12]). ip, intraperitoneal.

The ureagenesis assay was designed to provide a robust and physiologically meaningful measure of total urea cycle function across species and experimental systems without requiring challenging and not always informative tissue biopsies for enzymatic studies. In mice, the workflow is intentionally simple and minimally invasive (Figure 1), allowing repeated measurements and compatibility with a wide range of preclinical studies. The tracer, [^15^N]ammonium chloride, is administered intraperitoneally at a dose of 0.4–4 mmol per kilogram (kg) body weight (bw). This route ensures rapid systemic availability while avoiding the variability and risk associated with oral gavage. Following tracer administration, blood samples are collected at defined time points, typically at 30 and 60 min. Each sample requires only 5–10 μL of whole blood, which is immediately applied to filter paper and allowed to dry at room temperature. Once dried, the samples exhibit remarkable biochemical stability and can be stored either at ambient temperature for several days or at −20°C for extended periods without detectable loss of isotopic integrity [11]. Quantification of [^15^N]urea enrichment is subsequently performed using HR‐MS, which reliably distinguishes labeled from unlabeled metabolites based on their mass differences.

In human subjects, the assay follows a parallel conceptual framework but incorporates additional procedural safeguards appropriate for clinical research (Table 1). Participants undergo a 4‐h fasting period to minimize postprandial variability in nitrogen metabolism as well as delayed tracer passage and uptake. A peripheral venous catheter is placed to facilitate serial blood sampling with minimal discomfort. The tracer is administered orally at a dose of 2 mg/kg bw dissolved in a small volume (20–50 mL) of tap water. This tracer dose is 10 times lower of what was used in 1996 by Yudkoff et al. (20 mg/kg, 0.37 mmol/kg) and represents only around 0.55% of the total daily nitrogen allowance for a severe UCD patient [7, 12, 18]. Over the subsequent 2 h, six consecutive blood samples are obtained at predetermined intervals, allowing the establishment of an individualized ureagenesis time curve. To date, the procedure has been performed > 150 times in patients with UCDs and > 60 times in healthy controls, with excellent tolerability and no significant adverse events reported [12]. The combination of low tracer dose, short assay duration, and minimal invasiveness makes the method suitable for both pediatric and adult populations.

**TABLE 1:** Comparison of ureagenesis assay procedures in humans (proband or patient), in vivo and in vitro.

Situation	Requirements	References
Patient	Ethics approval according to local regulations (Study currently ongoing: NCT05671666) Fasting (minimum 4 h) Venous catheter installation Oral ^15^NH_4_Cl diluted in 20–50 mL water (2 mg/kg) Blood collection as dried blood spots (DBS) and/or plasma (each 100 μL) at 0; 15; 30; 45; 60; 90; and 120 min Sample shipment at room temperature (DBS) and/or on dry ice (plasma)	[12]
In vivo	Animal experimentation authorization according to nationals/locals laws Fasting for ≥ 2 h Intraperitoneal injection of ^15^NH_4_Cl (0.4–4 mmol/kg) Blood collection on filter paper as DBS at 0; 30; and 60 min (90 and 120 min) Sample shipment at room temperature	[11, 19, 20, 21, 22, 23, 24]
In vitro	Medium change to fresh medium with 1 mM of ^15^NH_4_Cl Supernatant collection at 24 h (≥ 100 μL) Sample shipment on dry ice	[25]

## 4 Advantages of This Ureagenesis Assay

A central strength of the here presented methodology lies in its ability to measure total urea cycle function, rather than relying on isolated metabolite concentrations that may be influenced by extrinsic factors or compensatory pathways. By directly tracing the incorporation of labeled nitrogen into urea, the assay captures the integrated activity of all enzymatic and transporter steps within the cycle, providing a complete assessment of pathway function. The minimal blood volume required, only a few microliters per time point, makes the assay particularly well suited for small animal models, neonatal studies, and repeated longitudinal measurements.

Another practical advantage is the exceptional stability of dried blood samples. This stability enables decentralized sample collection, facilitates multicenter studies, and allows shipment of samples by standard mail without the need for cold‐chain logistics.

The assay is also highly versatile. It can be performed not only in mice and humans but also in cellular systems (Figure 1b; Table 1), where the same tracer is added to culture medium and the incorporation of labeled nitrogen into urea is quantified in the supernatant [25, 26]. This flexibility allows direct comparison of ureagenesis capacity across primary hepatocytes, stem‐cell‐derived hepatocyte‐like cells, immortalized hepatic cell lines, and genetically modified cellular models.

Perhaps most importantly, the assay is sensitive enough to detect partial defects and subtle therapeutic responses that remain invisible to classical biomarkers such as ammonia, glutamine, or orotic acid [12]. Thus, the ureagenesis assay provides a physiologically grounded readout of metabolic capacity, making it a powerful tool for preclinical research, translational studies, and clinical monitoring.

## 5 Functional Assessment in *spf* ^ash^ Mice

Application of the ureagenesis assay to *spf* ^ash^ mice revealed a clear and reproducible functional distinction between mutant and wildtype animals, even under metabolically stable conditions in which classical biochemical markers fail to discriminate between genotypes. In wildtype mice, incorporation of the [^15^N]label into urea proceeded rapidly, with maximal enrichment observed approximately 30 min after tracer administration [13]. This early and robust labeling pattern reflects the high intrinsic capacity of the intact urea cycle to capture and detoxify ammonia immediately upon its arrival in the periportal hepatocytes.

In contrast, *spf* ^ash^ mice exhibited a markedly altered kinetic profile. The rise in [^15^N]urea enrichment was substantially delayed, and the initial rate of labeling was significantly reduced compared with wildtype controls [13]. Rather than achieving a rapid peak, mutant animals displayed a slow, gradual increase in labeled urea, reaching levels comparable to wildtype mice only after approximately 120 min. This prolonged time course is consistent with the known biochemical defect in OTC activity in *spf* ^ash^ mice and demonstrates the impaired ability of their urea cycle to process incoming nitrogen efficiently.

The 30‐min time point emerged as the most informative discriminator of genotype. At this early interval, non‐fasted *spf* ^ash^ mice achieved only ~33% of the ureagenesis observed in wildtype animals, providing a sensitive and quantitative measure of impaired urea cycle function. When animals were fasted prior to tracer administration, the functional deficit became even more pronounced, with mutants reaching only ~19% of wildtype enrichment at the same time point [11]. These findings highlight the influence of nutritional state on nitrogen metabolism and further underscore the sensitivity of the assay to physiological variables that modulate urea cycle activity. Importantly, the functional impairment revealed by the ureagenesis assay stands in stark contrast to the apparent biochemical normality suggested by classical metabolite measurements.

## 6 Application of the Ureagenesis Assay in Preclinical Studies

The ureagenesis assay proved particularly valuable in evaluating the efficacy of gene therapy approaches aimed at correcting UCDs by using different strategies. One illustrative example is its application in *Ass1* ^fold/fold^ mice, a hypomorphic model of citrullinemia type I characterized by markedly reduced argininosuccinate synthetase activity and chronically elevated plasma citrulline concentrations [27]. In these animals, in vivo AVV‐mediated gene addition or prime‐editing was employed to correct the underlying pathogenic variant. While reductions in plasma citrulline provided an initial indication of biochemical improvement, functional assessment of urea cycle function offered a physiologically meaningful measure of metabolic rescue [28].

Other examples of the usefulness of the ureagenesis assay are provided in preclinical studies applying diverse therapeutic approaches in a variety of UCD animal models [19, 20, 21, 22, 23, 24, 29].

## 7 Application of the Ureagenesis Assay in Cellular Models

To extend the utility of the ureagenesis assay beyond whole‐organism studies, the method was adapted for use in in vitro systems. In this setting, [^15^N]ammonium chloride is added directly to the culture medium, allowing labeled nitrogen to enter cellular metabolic pathways over a 24 h incubation period (Figure 1b). Analysis of culture supernatants revealed striking differences in ureagenesis capacity across various hepatocyte models, reflecting their intrinsic metabolic maturity and urea cycle competence [25].

Primary human hepatocytes exhibited the highest functional activity, with more than 35% of total urea pool labeled after tracer exposure. In contrast, induced pluripotent stem cell (iPSC)‐derived hepatocytes, despite expressing key hepatic markers, demonstrated only little ureagenesis, with labeling levels around 5%. HepaRG cells, a commonly used hepatic cell line with confirmed urea cycle functionality, showed intermediate performance at approximately 8% enrichment [25]. These results underscore the assay's ability to discriminate between cell types based on functional metabolic output rather than solely on gene expression profiles or surrogate markers of differentiation. As such, the ureagenesis assay provides a powerful tool for evaluating hepatocyte maturation protocols, validating engineered cell lines, and assessing the metabolic consequences of in vitro genetic manipulation.

## 8 Discussion

A central finding of this work is that classical metabolite measurements, although indispensable for the diagnosis and acute management of UCDs, are fundamentally limited in their ability to reflect the true pathway function. Metabolite concentrations represent the steady‐state balance between production, utilization, and excretion. In the context of UCDs, this balance can be profoundly influenced by factors that are independent of the underlying molecular defect. Residual enzyme or transporter activity, even when markedly reduced, may be sufficient to maintain near‐normal metabolite levels under basal conditions. Dietary protein intake and microbiome, which modulates nitrogen load [30, 31], can mask or exaggerate biochemical abnormalities depending on timing and composition. Renal clearance further contributes to variability, as the kidneys play a relevant role in nitrogen disposal and can compensate for hepatic insufficiency [32]. In addition, a variety of compensatory metabolic pathways, including glutamine synthesis, amino‐acid transamination and alternative nitrogen‐handling routes provided by scavengers [16, 33], can normalize circulating metabolites despite an impaired urea cycle.

These limitations underscore the importance of functional pathway analysis, which provides a fundamentally different type of information. The ureagenesis assay measures total pathway function, not single metabolite concentration. This distinction is critical: the assay reflects the actual capacity of the urea cycle in real time, whereas metabolite concentrations reflect only the actual situation of single players of the pathway. By directly quantifying the incorporation of labeled nitrogen into urea, the ureagenesis assay captures the integrated activity of all steps within the urea cycle. This approach is inherently more sensitive to partial defects, subtle improvements, and regional differences in hepatic function, and it provides a more accurate assessment of therapeutic interventions than metabolite measurements.

The superiority of this method becomes even more apparent when compared with earlier tracer‐based approaches, such as those employing [^13^C]acetate [34, 35, 36] (Table 2) or [^15^N]glutamine and [^18^O]urea [37]. Although [^13^C]acetate has been used to assess ureagenesis using isotope ratio mass spectrometry, its physiological properties limit its utility. Hepatic first‐pass extraction of [^13^C]acetate is extremely low, typically < 1%, meaning that the majority of the tracer bypasses the liver with a large fraction (> 98%) exhaled as ^13^CO_2_ in breath. This results in substantial tracer loss, necessitating concomitant breath analysis and complicating interpretation. Moreover, the [^13^C]acetate method allows quantification only of labeled urea, without providing information on intermediate metabolites or parallel nitrogen‐handling pathways [36]. The inability to use dried blood spots further restricts its practicality. In contrast, [^15^N]ammonium chloride exhibits hepatic first‐pass extraction exceeding 50%, ensuring that the tracer is delivered directly to the periportal hepatocytes. This physiological advantage, combined with the analytical precision of HR‐MS, makes [^15^N]‐based ureagenesis testing a superior tool for studying urea cycle function.

**TABLE 2:** Comparative summary of diagnostic methods using metabolites or tracer studies for urea cycle disorders.

	Metabolites	[^13^C]Acetate	[^15^N]Ammonium chloride
Advantage	Available to most clinical centers Informative in decompensation Fast turnaround time Does not require specific procedures	Precise mass spectrometry approach Validated in controls and in patients affected by OTC deficiency	Precise mass spectrometry approach Validated in controls and in patients affected by UCDs, in vivo and in vitro Suited for clinical diagnostic, therapeutic approaches assessment, and preclinical studies
Limitation	Single timepoint analysis Not informative in compensated states Dependent on external factors Complex interpretation	Large fraction of tracer lost in breath Complex analysis and interpretation Currently not widely available (isotope ratio MS)	Complex sample and data analysis Salty tracer taste Currently available only in a single specialized lab

*Note:* Metabolites: ammonia, amino acids in plasma, and orotic acid in urine.

Abbreviations: OTC, ornithine transcarbamylase; UCDs, urea cycle disorders.

The assay's versatility enables its application across a broad range of preclinical research settings. In gene addition and gene editing studies, it provides a sensitive and quantitative measure of metabolic rescue, capturing improvements that may not be reflected in classical biomarkers or in the assessment of enzymatic activities obtained from liver biopsy. It is equally valuable for characterizing new animal models, particularly those with hypomorphic or tissue‐specific defects that produce subtle biochemical phenotypes. In the field of hepatocyte biology, the assay offers a functional readout of cellular maturity, enabling rigorous evaluation of differentiation protocols for stem‐cell‐derived hepatocytes or engineered hepatic cell lines. Beyond inherited metabolic disorders, the method has potential applications in cancer biology, where nitrogen metabolism is frequently rewired toward pyrimidine synthesis [38], and in immunology, where urea cycle intermediates influence immune cell function [39]. Its ability to demonstrate metabolic stability without requiring invasive procedures also makes it a powerful tool for chronic disease modeling.

In clinical research, the ureagenesis assay fills a critical gap left by traditional biomarkers. In patients with compensated UCDs and with mutations of uncertain significance, metabolite levels often fall within the normal range, obscuring meaningful differences in metabolic capacity. Functional ureagenesis testing enables accurate assessment of urea cycle function in these individuals, providing insight into disease severity that cannot be obtained from a snapshot of metabolite concentrations. It also offers a sensitive means of evaluating therapeutic efficacy, particularly for emerging treatments such as gene therapy, mRNA therapy, and small molecule modulators, where improvements may be partial or gradual. By quantifying whole pathway function, the assay supports more precise stratification of disease severity and facilitates monitoring of the long‐term outcome of a procedure by assessing the metabolic situation without the need for invasive procedures. As the therapeutic landscape for UCDs continues to expand, functional assays such as this one will become essential tools for clinical trials and personalized medicine, enabling more accurate evaluation of treatment response and more informed clinical decision‐making. Accordingly, the ureagenesis assay has already become part of an ongoing clinical trial (ClinicalTrials.gov NCT06488313), in which it serves as a secondary outcome measure.

As a practical limitation, the ureagenesis assay requires HR‐MS that is only available in few centers. In addition, the unpleasant taste of [^15^N]ammonium chloride may reduce patients' compliance although it has been shown to be safe and not to increase blood ammonia concentrations [12].

In conclusion, classical biochemical markers provide limited insight into urea cycle function, particularly in compensated states or during therapeutic interventions. This [^15^N]ammonium chloride‐based ureagenesis assay offers a robust, sensitive, and physiologically relevant measure of total urea cycle function. It is applicable across species and experimental systems, from mice to humans to hepatic‐like cells, and has demonstrated utility in several preclinical studies, cellular models, and in clinical evaluation.

By quantifying dynamic metabolic function rather than a snapshot of metabolite concentrations, this assay overcomes the limitations of traditional biomarkers and provides a powerful tool for preclinical and clinical research in UCDs.

## Funding

This study was supported by the Swiss National Science Foundation (grants 320030_207965 and CRSII‐222794), University Research Priority Program ITINERARE, and Citrin Foundation (grant reference number: RG24003).

## Disclosure

A European patent application (No. 25168775.2) has been filed for the ureagenesis assay described in this manuscript.

## Ethics Statement

The authors have nothing to report.

## Consent

The authors have nothing to report.

## Conflicts of Interest

The authors declare no conflicts of interest.

## Data Availability

Data sharing not applicable to this article as no datasets were generated or analyzed during the current study.
